# Antibacterial activity of a sterile antimicrobial polyisoprene surgical glove against transient flora following a 2-hours simulated use

**DOI:** 10.1186/s12893-015-0058-5

**Published:** 2015-07-04

**Authors:** Johannes Leitgeb, Rupert Schuster, Bit New Yee, Pui Fong Chee, Julian-Camill Harnoss, Peter Starzengruber, Michael Schäffer, Ojan Assadian

**Affiliations:** Department for Trauma Surgery, Medical University of Vienna, Waehringer Guertel 18-20, A-1090 Vienna, Austria; Science & Technology Innovation Centre, Ansell Shah Alam, 40000 Shah Alam, Selangor Malaysia; Department of General, Visceral and Transplant Surgery, University Hospital Heidelberg, Im Neuenheimer Feld 110, D-69120 Heidelberg, Germany; Department for Hospital Hygiene, Medical University of Vienna, Waehringer Guertel 18-20, A-1090 Vienna, Austria; Department for General, Visceral, and Thoracic Surgery, Marienhospital Stuttgart, Böheimstrasse 37, D-70199 Stuttgart, Germany; Institute for Skin Integrity and Infection Prevention, School of Human & Health Sciences, R1/29 Ramsden Building, University of Huddersfield, Huddersfield, HD1 3DH UK

**Keywords:** Antimicrobial, Surgical glove, Transient flora, Antibacterial efficacy, Suppression

## Abstract

**Background:**

A surgical glove will protect surgeons and patients only if the glove’s integrity remains intact. However, several studies have demonstrated that undetected micro-perforations of surgical gloves are common. Because of the possibility of surgical glove puncture, an antimicrobial surgical glove was developed. The aim of this laboratory based experimental study was to assess the antibacterial efficacy of the interior chlorhexidine-gluconate (CHG)-coat of an antimicrobial synthetic polyisoprene surgical glove by using a standardized microbiological challenge.

**Methods:**

Sixteen healthy adult participants donned one antimicrobial surgical glove and one non-antimicrobial surgical glove randomly allocated to their dominant and non-dominant hand following a crossover design. During a 2-h wear time, participants performed standardized finger and hand movements. Thereafter, the interior surface of excised fingers of the removed gloves was challenged with 8.00 log_10_ cfu/mL *S. aureus* (ATCC 6538) or *K. pneumoniae* (ATCC 4352), respectively. The main outcome measure was the viable mean log_10_ cfu counts of the two glove groups after 5 min contact with the interior glove’s surface.

**Results:**

When comparing an antimicrobial glove against an untreated reference glove after 2-h simulated use wear-time, a mean reduction factor of 6.24 log_10_ (*S. aureus*) and 6.22 log_10_ (*K. pneumoniae*) was achieved after 5 min contact.

**Conclusion:**

These results demonstrate that wearing antibacterial gloves on hands does not negatively impact their antibacterial activity after 2-h of wear. This may have a potential benefit for patient safety in case of glove puncture during surgical procedures.

## Background

The intact surgical glove serves as an important barrier against bi-directional migration of micro-organisms between the hands of the surgical team members and the surgical site [1, 2]. However, a surgical glove will protect both only if the glove’s integrity remains intact. Several studies have demonstrated that undetected micro-perforations of surgical gloves are common [3, 4]. The risk of glove defects increases with time of wear [5] and is related to the type of surgery performed, ranging from 7 % in urological surgery and 65 % in cardio-thoracic surgery [6–10].

Because of the risk of surgical glove puncture, a sterile powder free antimicrobial surgical glove was developed. The glove is coated on its inner surface with a complex of anti-irritants, moisturizers, emollients, and chlorhexidine digluconate (CHG) as the active antimicrobial compound. The glove may be worn by health care workers during surgical procedures and is intended to protect them from blood-borne microorganisms and pathogens originating from infected surgical sites in case of a glove breach. Percutaneous injuries occur regularly during surgery, placing surgical personnel at risk for localized and systemic infection with pathogens originating from the patient. The overall frequency of percutaneous blood and body fluid exposures in operating rooms ranges from 2.3 to 3.8 exposures per 100 surgical procedures. [11] Such exposures may be associated with transmission of blood borne pathogens, notably Hepatitis B virus (HBV), Hepatitis C virus (HCV), and Human Immunodeficiency virus (HIV). Overall, for HBV, HCV, and HIV a surgeon’s cumulative lifetime risk of acquiring HBV, HCV, and HIV infection in low-prevalence regions was estimated to be 43 %, 35 %, and 0.5 %, respectively. [12] However, the risk of occupational percutaneous injuries shows geographic variability, with up to 10.5 exposures per 100 surgical procedures in developing regions. [13] Additionally, the frequency depends on the surgical operation technique, ranging from 4.3 exposures per 100 in laparotomic abdominal procedures to 1.3 exposures per 100 vascular surgical procedures, and 1.1 exposures per 100 maxillofacial surgical procedures [14]. Furthermore, it was observed that the exposure frequency increases with an increase in estimated patient blood loss (1.8 exposures per 100 procedures), increased number of surgical personnel working in the surgical field (2.1 exposures per 100 procedures), and increased duration of surgical procedures. For surgical procedures lasting 4–6 h, the reported frequency is 1.4 exposures per 100 procedures, while for procedures lasting longer than 6 h the frequency increases to 2.4 exposures per 100 procedures. [15] While antimicrobial gloves do not reduce the risk of glove puncture or percutaneous injury, such concepts are believed to kill or inactivate potential pathogens entering the space between the inner side of the punctured glove and the skin of healthcare workers, or to reduce microbial passage across punctured surgical gloves. [16]

However, contrary to this indented use, recently it was demonstrated that the concept of such an antimicrobial surgical glove is able to suppress the wearer’s bacterial hand flora during the course of vascular surgical procedures [17]. In this randomized controlled study, the difference of recovered number of bacterial hand flora between antimicrobial and standard surgical gloves was 1.30 log_10_ cfu/mL, and statistically significant (p < 0.001). Yet, the design of this trial did not allow exact measurement of the glove’s total antibacterial reduction efficacy, as the participating surgeons performed a surgical hand rub before donning. Hence, the reduction factor of 1.30 log_10_ represented only the difference in re-growth of the normal skin flora during 112 min of operation time. In order to assess the antibacterial efficacy of a CHG-coated synthetic polyisoprene surgical glove even under controlled condition, this laboratory-based experimental study was conducted.

## Methods

### Experimental setting

This study involved 16 healthy adult participants. Participants were employees of the glove manufacturer, working and handling the investigated gloves on a regular basis. Since no specimens or personal identifiable biometric information was collected from the participants, this study did not require ethical committee approval according to the Medical Research and Ethics Committee (MREC), Ministry of Health Malaysia. All participants were informed about the composition of the investigated gloves and gave their consent to participate. Participants tested both, one antimicrobial surgical glove (intervention glove; CHG-coated sterilized Gammex PF Synthetic Polyisoprene Antibacterial surgical glove; Ansell Ltd., Richmond, Australia) and one non-antimicrobial surgical glove (reference glove; uncoated sterilized Gammex PF Synthetic Polyisoprene; Ansell Ltd., Richmond, Australia) randomly allocated to their dominant and non-dominant hand. The experiments were performed in duplicate following a cross-over design as such, that half of the participants tested once the intervention glove on the dominant hand and the reference glove on the non-dominant hand, and the other half vice versa. Thereby, 16 participants provided paired results for the intervention and the reference glove.

In total, 16 pairs of antimicrobial surgical gloves and 16 pairs of sterilized non-antimicrobial surgical gloves were used for this study exploring performance against 2 test microorganisms. The main outcome measure was the viable mean log_10_ cfu counts of a high bacterial inoculum after 5 min contact with the interior glove’s surface following a 2-h simulated glove wear. Prior to donning gloves, participants applied 3 mL of an alcohol-based hand rub (Softa-Man® - 45 % w/v Ethanol/18 % w/v Propanol; B.Braun, Melsungen, Germany), dispensed it into the palm of each hand and rubbed hands across all fingers and palms until dry. After hands became completely dry, all participants donned intervention and reference gloves as described and left the gloves on their hands for a 2-h wear time.

### Hand motion during testing

In order to simulate mechanical stress and hand movements typically found during surgical procedures, all participants performed a pre-exercised and standardized movement routine. In the first hour of wear, participants were required to type an 800-word article [18] on a personal computer, ensuring at least 400x movements for each hand [19, 20]. To further promote perspiration on the participants' hands, the second hour of the hand motion procedure involved twisting a l-kg ceramic former by turning it 100x at 5 workstations for 360° clockwise and anti-clockwise.

### Sampling and microbiological processing

At the completion of the 2-h wearing time, gloves were carefully removed by laboratory staff and placed on a designated collection tray without mixing CHG-coated and uncoated gloves. Three middle glove fingers of each glove were excised and filled with 0.1 mL of challenge suspension containing 8.00 log_10_ cfu/mL *Staphylococcus aureus* (ATCC 6538) or *Klebsiella pneumoniae* (ATCC 4352). For gloves size 6.0, the average glove finger length was 5.9 cm, for size 7.0 it was 5.3 cm, for size 7.5 the length was 5.0 cm, and for size 8.5 the average length was 4.7 cm. In each glove finger challenge bacteria were spread by gentle massage to allow contact with the entire glove’s interior surface during 5 min contact time. Thereafter, 0.9 mL of BBP++ (Butterfield’s Buffered Phosphate stock solution with a validated neutralizer based on 10 % Tween 80 and 1 % lecithin, and active against CHG [21]) was added. Serial dilutions were prepared and final samples were plated on Tryptic soy agar (TSA) plates (Merck KGaA, Darmstadt, Germany), and incubated for 48 h at 37 °C ± 1 °C.

### Statistical analysis

After incubation, visible colony forming units (cfu) were counted and recorded for each dilution step. The number of cfu per mL sampling fluid was calculated by multiplying the plate count by the dilution factor. Counts were obtained from plates growing 30 to 300 cfu. For both, reference and intervention glove, viable counts were transformed to decimal logarithms (log_10_), averaged separately, and the arithmetic means of all individual log_10_ reduction values were calculated.

The number of subjects was determined to allow a statistical power of 80 % (alpha = 0.05). Sample size calculation showed that a sample size of 16 human participants would be sufficient for an accepted confidence level of 95 % at an expected data’s standard deviation of 0.4 and an accepted sampling error of 20 %.

For continuous variables, means ± standard deviation (±SD) were calculated. Mean log_10_ cfu/mL counts were tested for statistical significant difference by using the two-sided Mann–Whitney U test. The confidence level was set at 95 %, and a P-value of < 0.05 indicated statistical significant difference in the post-values of the yielded numbers of test organisms between the reference and the intervention glove. The study’s test hypotheses were:

H_0_: P_R_ = P_I_

H_A_: P_R_ ≠ P_I_where

P_I_ = Median microbial population recovered from the intervention glove, and

P_R_ = Median microbial population recovered from the reference glove

## Results

Table 1 summarizes the results for the mean log_10_ cfu recovery of *S. aureus* (ATCC 6538) or *K. pneumoniae* (ATCC 4352) after 5 min contact time following wearing of the intervention and reference gloves for 2 h. For *S. aureus*, the CHG-coated synthetic polyisoprene surgical intervention glove demonstrated a mean reduction factor of 6.24 log_10_, when compared against the uncoated reference glove (P < 0.001).

**Table 1:** Mean log_10_ cfu recovery of *S. aureus* or *K. pneumoniae* at 5 min contact time after 2-h glove wear time

	*S. aureus*		*K. pneumoniae*	
	Control glove	Test glove	Control glove	Test glove
Sample size	16	16	16	16
Mean log_10_ cfu recovery ± SD	7.37 ± 0.09	1.12 ± 0.23	7.28 ± 0.12	1.05 ± 0.10
Min.	7.23	1.00	7.05	1.00
Max.	7.56	1.73	7.43	1.36
Mean log_10_ reduction ± SD	6.24 ± 0.24		6.23 ± 0.12	
P-value	<0.001		<0.001	

For *K. pneumoniae*, the intervention glove showed a mean reduction factor of 6.22 log_10_ after 5 min contact time when compared against the untreated reference glove after the 2 h simulated use wear-time (P < 0.001).

## Discussion

This study demonstrated that an antibacterial surgical glove has an efficacy of more than 6 log_10_ in killing transient microorganisms such as *S. aureus* or *K. pneumoniae* within 5 min contact time following a 2-h wear time.

Before starting a surgical procedure, surgeons do not done gloves with any transient microbial bio-burden on the hands below the sterile surgical gloves. However, before this experimental study, it was not fully clear if the concept of an antimicrobial surgical glove with an internal CHG-coating is also able to be effective against immediate introduction of a high bacterial inoculum after a longer wear time. Because of practical reasons, our study protocol included a 2-h wear time during which standardized hand and finger movement were performed before an extreme bacterial bio-burden was inoculated inside the gloves. The mean duration of most surgical procedures usually ranges around 170 min of procedure time [22], with wide variations. However, it was also demonstrated that after 90 min of operating time, approximately 20 % of surgical gloves are perforated [5]. Furthermore, one pair of surgical gloves will rarely be worn without changing for longer than 2 h. After this time, most surgeons will naturally have donned their hands with a new pair of surgical gloves. Therefore, and because the duration of 42 % of all reported surgical procedures last 2 h or shorter [22], it was feasible and more practically oriented to evaluate the intervention glove’s antimicrobial efficacy after a 2-h wear time.

Our study has a number of limitations. First, because this study was not designed to assess possible skin reactions or adverse events during or after the experiments, we cannot elaborate on such possible adverse outcomes. Second, whilst the hand movements performed in our study did not directly mimic a specific surgical procedure, they tried to provide a realistic simulation in terms of mechanical stress. There is little published on hand movements during surgical procedures. The available literature, however, gives some interesting insights into laparoscopic procedures. Two published papers were found that describe the number of hand movements in the operating room [19, 20] using a motion analysis/tracking device (Imperial Collage Surgical Assessment Device; Imperial College, UK). Dosis et al. [19] investigated synchronized hand kinematics and video from real 10 laparoscopic cholecystectomies performed by 5 different surgeons. The analysis focused on the entire procedure and also on specific parts of the operation such as clipping and cutting of the cystic duct and artery. During the extraction and insertion of a scissors-stapler instrument over 201 s, the left hand was moved 240 and the right hand 380 times. For the clipping and cutting of the cystic duct, 13 – 17 s were spent, with 29 – 32 movements for the right hand. These results indicate that during high-velocity phases of a surgical procedure, hands are moved faster than 1x per second, and that the activity of the right hand is higher than of the left hand. Regretfully, the authors did not indicate if all right hands were dominant, and all left hands non-dominant hands of participating surgeons. However, the more experienced a surgeon was, the fewer movements per second were recorded. Even in this controlled setting, hand movements showed a wide variability between surgeons. Aggarwal et al. [20] used the same motion tracking system, but this time in 16 experienced and inexperienced surgeons operating laparoscopically on 53 patients with a diagnosis of biliary colic. Again, wide variations between duration and number of movements were observed, including differences in dominant and non-dominant hands.

Common patterns for hand movements during surgery seem to be changing sequences of no hand movements, slow, fast, and high-velocity hand movements. Based on the available literature, standardized hand movements were used in our study to attempt to mimic the number of hand movements and the alteration in high velocity seen in the published studies. While the implemented hand movements may be viewed as a limitation of our study, as they may not be representative for a broader scope of surgical procedures, there are no compelling facts which indicate that hand movements during office or laboratory work may not be adequately sufficient to allow drawing conclusions for the situation in an operation room. However, because the available literature indicates that a difference between the movement activity of the right and left hands exists during surgical procedures, our study was specifically executed where reference and intervention gloves were distributed evenly among the participants’ dominant and non-dominant hands.

## Conclusion

The results of this study demonstrate that antibacterial gloves worn on hands after 2-h of wear are able to achieve a mean reduction factor of 6.24 log_10_ (*S. aureus*) and 6.22 log_10_ (*K. pneumoniae*) after 5 min contact time. Furthermore, such wear time does not negatively impact their antibacterial activity. This may have a potential benefit for patient safety in case of glove puncture during surgical procedures.

## Abbreviations

ATCC: American Type Culture Collection (Manassas, VA, USA)
CHG: Chlorhexidine Digluconate
cfu: colony-forming unit
Gr+: Gram-positive bacteria
Gr-: Gram-negative bacteria
H_0_: Null-hypothesis
H_A_: Alternative hypothesis
HPLC: High performance liquid chromatography
MBC: Minimal bactericidal concentration
TSA: Tryptone soya agar
